# Precision Dopaminergic Treatment in a Cohort of Parkinson’s Disease Patients Carrying Autosomal Recessive Gene Variants: Clinical Cohort Data and a Mini Review

**DOI:** 10.3390/neurolint16040062

**Published:** 2024-07-30

**Authors:** Christos Koros, Athina-Maria Simitsi, Nikolaos Papagiannakis, Anastasia Bougea, Roubina Antonelou, Ioanna Pachi, Evangelos Sfikas, Evangelia Stanitsa, Efthalia Angelopoulou, Vasilios C. Constantinides, Sokratis G. Papageorgiou, Constantin Potagas, Maria Stamelou, Leonidas Stefanis

**Affiliations:** 1 1st Department of Neurology, Eginition Hospital, National and Kapodistrian University of Athens, 11528 Athens, Greece; simitsh@yahoo.gr (A.-M.S.); nickpap88@gmail.com (N.P.); annita139@yahoo.gr (A.B.); rantonelou@gmail.com (R.A.); pachiioanna@gmail.com (I.P.); vasfikas@gmail.com (E.S.); eva.st.92@gmail.com (E.S.); angelthal@med.uoa.gr (E.A.); vassilis.kon@hotmail.com (V.C.C.); sokpapa@med.uoa.gr (S.G.P.); cpotagas@gmail.com (C.P.); lstefanis@bioacademy.gr (L.S.); 2 Hygeia Hospital, 15123 Athens, Greece; mariastamelou@gmail.com

**Keywords:** Parkinson’s disease, genetic, recessive, treatment, levodopa, dopamine agonists, amantadine

## Abstract

Introduction: Parkinson’s disease (PD) patients harboring recessive gene variants exhibit a distinct clinical phenotype with an early disease onset and relatively mild symptoms. Data concerning individualized therapy for autosomal recessive PD forms are still scarce. Methods: Demographic and treatment data of a cohort of PD carriers of recessive genes (nine homozygous or compound heterozygous *PRKN* carriers, four heterozygous *PRKN* carriers, and three biallelic *PINK1* carriers) were evaluated. Results: The average levodopa equivalent daily dose (LEDD) was 806.8 ± 453.5 (range 152–1810) in *PRKN* carriers and 765 ± 96.6 (range 660–850) in *PINK1* carriers. The majority responded to low/moderate doses of levodopa. The response to dopamine agonists (DAs) was often favorable both as initial and longitudinal therapy. In total, 8/13 *PRKN* and 1/3 *PINK1* carriers were treated with amantadine successfully, and this also applied to patients who could not tolerate levodopa or DAs. Conclusions: In the era of personalized treatment, the therapeutic approach in recessive PD gene carriers might differ as compared to idiopathic PD. Lower LEDD doses were efficient even in patients with a very long disease duration, while a few patients were doing well without any levodopa treatment decades after disease initiation. DAs or amantadine could be used as a first and main line treatment regimen if well tolerated. Literature data on therapeutic strategies in carriers of pathogenic mutations in recessive PD genes, including device-aided treatments, will be further discussed.

## 1. Introduction

The genetic background could likely explain a good part of the heterogeneity ob-served in progressive motor and cognitive decline among Parkinson’s disease patients (PD). In monogenic forms of PD, specific mutations correspond to a defined pattern of clinical impairment. The stronger the association between the genotype and alpha-synuclein pathology, the greater the risk for severe motor and non-motor symptomatology. Interestingly, PD patients harboring recessive gene variants exhibit a distinct clinical phenotype with an early disease onset (EOPD) and relatively mild symptoms.

Mutations in the gene encoding for Parkin (*PRKN*) are the most commonly identified genetic cause of EOPD [1,2]. The genetic deficit can be missense/nonsense mutations or a copy number variant (CNV) causing loss of function. Parkin is an E3 ligase, participating in the degradation of specific substrates through the ubiquitin-proteasome system. Its ex-act role in PD has yet to be clarified, but the evidence suggests the involvement of Parkin at the level of mitochondria, in the process of the proper removal of damaged mitochondria through mitophagy [3]. In terms of pathology, *PRKN*-related PD is a nigropathy and the most prominent finding is the degeneration of neurons in the brainstem nuclei (the substantia nigra pars compacta (SNpc) and the locus coeruleus are pigmented, but the raphe nuclei, which also degenerate in PD, are not) [4]. Notably, the loss of neurons was less prominent in the locus coeruleus than in the substantia nigra pars compacta as is also the case in idiopathic PD. Lewy bodies were absent in the majority of cases and only in the post-mortem assessments of three patients have researchers verified the presence of Lewy bodies with alpha-synuclein depositions. A few other affected individuals had neurofribrillary tangles (Tau pathology) [5]. The phenotype of *PRKN* variants is characterized by a benign course with slow progression, a favorable response to levodopa or anticholinergic medications, common presentation as dystonia, especially in the lower extremities, and the frequent emergence of dyskinesias and motor fluctuations. Non-motor symptoms are less prominent than in idiopathic PD. Sleep benefit has been reported, and exercise-induced dystonia may be present, resulting in a rather demanding clinical differential diagnosis with dopa-responsive dystonia (DRD) [1,6]. Psychiatric manifestations like anxiety, depression, obsessive-compulsive disorder-like symptoms, and even frank psychosis might occur, sometimes antedating motor symptoms. Cognition is not affected even after a very long disease duration and patients often perform better as compared to idiopathic PD [7,8]. Notably, imaging with DATSCAN SPECT in *PRKN* carriers reveals impaired dopaminergic innervation to the caudate nucleus even to a greater extent than in idiopathic PD [1].

Whether heterozygote Parkin mutations result in a PD phenotype is still elusive [9]. The importance of heterozygous mutations is rather controversial and could be explained by a gain of function or a negative effect, haploinsufficiency, and concomitant difficult-to-identify mutations either within the Parkin gene (in trans) or within other genes. Addi-tional genetic, epigenetic, or environmental factors might play a role in the clinical im-portance of heterozygous *PRKN* mutations. A recent study assessed how monoallelic or biallelic pathogenic variants in the *PRKN* gene may affect its transcription in peripheral blood mononuclear cells (PBMCs). A significant decrease in *PRKN* mRNA expression lev-els was observed in both heterozygous and biallelic *PRKN* PD carriers as compared to idiopathic PD and healthy controls [10]. Another study has shown a significant risk for PD only for heterozygote dosage variants and not for point mutation heterozygote carriers [11]. It is possible that in certain early-onset PD patients, heterozygous dosage *PRKN* mu-tations might play a causal role while in late-onset PD such mutations could be an inci-dental finding [2].

After Parkin, mitochondrial PTEN (phosphatase and tensin homologue)-induced ki-nase1 (*PINK1*) mutations appear to represent the second most common cause of EOPD. Missense, nonsense, splice mutations, or small deletions or insertions are encountered, either in a compound heterozygote or homozygous state. *PINK1* is a kinase localized to the mitochondria and the mechanism of the disorder is considered to be a loss-of-function mitochondrial disease. Its exact role remains uncertain, but it appears to be involved in the pathway of mitophagy, acting upstream of Parkin. Furthermore, it may also have a role in the proper function of mitochondrial complex I [12]. Regarding pathology in *PINK1*-related PD, the literature data are scarce [4]. An autopsy in a compound heterozygote showed neuron loss in the substantia nigra pars compacta while the locus coeruleus was spared. Lewy body pathology was present and degeneration was also evident in the nucleus basalis of Meynert [13]. The phenotype involves a relatively early age of onset, a rather benign course, more frequent gait deficits and disease onset with lower limb problems, excellent response to levodopa therapy, and increased risk for dyskinesias. Notably, dementia is not typical of *PINK1* patients even after a long disease duration although cognitive deficits may occur in some patients [2,7]. As far as other non-motor symptoms are concerned, patients with *PINK1* mutations often have decreased olfaction [14] while some may also manifest autonomic dysfunction [15]. Despite few previous studies, REM sleep behavior disorder is rarely described, even in advanced cases [8,16]. Anxiety, depression, panic attacks, or psychosis have been reported in certain cases and may even antedate motor symptoms [12,17].

An additional rare autosomal recessive form of EOPD is due to mutations in the Pro-tein deglycase *DJ-1* gene, encoding for a protein which has a role in the antioxidant re-sponse and may participate in common biochemical pathways with *PINK1* and *PRKN* [3]. The *DJ-1* phenotype resembles that usually seen in Parkin-related PD including an early age of onset, slow progression, frequent occurrence of focal dystonia, and a good response to dopaminergic treatment but often prominent motor complications upon treatment, variable cognitive involvement, and psychiatric symptoms, especially anxiety [2,7].

The aim of the present study was to assess the dopaminergic therapy requirements of a cohort of PD patients carrying pathogenic mutations in recessive genes (*PRKN*, *PINK1*) and its outcome in terms of motor and non-motor competence of patients. Moreover, the current literature data on conventional and device-aided treatments in this particular PD patient population will be reviewed.

### Literature Data on Autosomal Recessive PD Device-Aided Therapies

Previous studies based on data derived from case series have shown an overall fa-vorable response to levodopa treatment; however, literature evidence concerning individ-ualized therapy for autosomal recessive PD forms are still sparse. The majority of past re-search on PD carriers of recessive genes has focused mostly on device-aided therapies in advanced stages of the disorder [18,19,20]. Genetic PD variants with a full-blown phenotype which includes motor complications, psychiatric problems, cognitive deficits, impulse control disorders, and peripheral neuropathy may require different therapeutic strategies. *PRKN* mutation carriers generally are considered to have a favorable response to deep brain stimulation (DBS) treatment. In most studies, motor amelioration in the *PRKN* car-rier group was similar when single heterozygous *PRKN* mutations were excluded. A moderate or even poor response to DBS could be attributed to a non-favorable initial re-sponse to L-Dopa prior to surgery in certain patients, to a more pronounced axial symp-tomatology, and occasionally to improper target selection [20]. A study by Moro and co-authors [21] showed that following bilateral subthalamic nucleus (STN) DBS surgery, in 11 *PRKN* mutation carriers (6 biallelic and 5 monoallelic), both mutation carriers and non-carriers exhibited an approximately 42% improvement in motor scales including Unified Parkinson’s Disease Rating Scale part III (UPDRSIII) 3–6 years post treatment. However, it was clear that the response to DBS was not superior in *PRKN* carriers compared to non-carriers and could be limited by more advanced axial motor symptomatology at a rather early disease stage [21]. In another cohort, nine *PRKN* mutation carriers (four biallelic and five monoallelic) patients underwent surgery with either bilateral STN DBS or bilateral Globus Pallidus Internus (GPi) DBS. STN DBS resulted in a greater decrease in motor scores, while GPi DBS patients exhibited a significant improvement in dyskinesias [22]. Lohmann and co-authors (2008) assessed a cohort which included 14 *PRKN* mutation carriers who underwent bitaleral STN DBS. Motor and non-motor scores were comparable between *PRKN* carriers and sporadic PD; however, the levodopa equivalent dose (LEDD) was lower in *PRKN* carriers post surgery [23]. Similarly, Kim and co-authors did not report differences in motor and non-motor scores between *PRKN* carriers and non-carriers but noted a 70% reduction in LEDD [24]. It appears that axonal symptoms like postural stability were worse in *PRKN* carriers than in non-carriers following STN DBS [24]. DBS is probably a safe option in terms of cognitive deterioration in PD patients carrying *PRKN* mutations. Moreover, there are insufficient data regarding the impact of the type of *PRKN* mutations on the clinical outcome following DBS [19]. Continuous apomorphine subcutaneous infusion in *PRKN* was published as a case report and a marked improvement in dyskinesias and OFF time was reported. Moreover, the overall mobility was better and the falls became scarce. Oral dopaminergic medication could be decreased [25]. Finally, to our knowledge, there are limited data on levodopa/carbidopa intestinal gel infusion (LCIG) in *PRKN* mutation carriers, with only one case reported [26].

Studies on device-aided therapies in *PINK1* carriers are scarce and have only includ-ed case reports due to the rarity of carriers. Moro and co-authors [21] reported a 40% im-provement in UPDRSIII score following STN DBS which was sustained in follow-up. An-other case report showed a significant improvement in fluctuations and other motor symptoms. Impulse control disorder was ameliorated post treatment and non-motor symptoms were relatively stable with the exception of hypersomnolence [26]. Finally, Bo-rellini and co-authors reported that after bilateral GPi DBS in a *PINK1* carrier, motor com-plications were improved but there was an increase in freezing 4 years post surgery [27,28]. No data on the outcome of LCIG or continuous apomorphine infusion regarding pathogenic *PINK1* mutation carriers are available in the literature.

## 2. Materials and Methods

We assessed a cohort of PD carriers of pathogenic mutations in recessive genes: 9 were homozygous or compound heterozygous *PRKN* carriers (pathogenic mutations), 4 heterozygous *PRKN* carriers, and 3 biallelic *PINK1* carriers (Mendelian inheritance in man [MIM] numbers * 602544 and * 608309, respectively, An Online Catalog of Human Genes and Genetic Disorders, https://www.omim.org/), while no carrier of *DJ-1* pathogenic mutations could be identified. *PRKN* mutations included both point and dosage mutations. The co-existence of mild and/or severe Glucocerebrosidase (*GBA1*) gene mutations had been previously excluded. The patients mentioned above were followed in the Movement Disorders Outpatient clinic of Eginition Hospital, NKUA, and have been enrolled in the ‘’Thalis’’ database and biobank.

Demographic [age, sex, age at disease onset, and disease duration) and basic clinical data (including motor disability grading with the Hoehn and Yahr Scale (H&Y), the pres-ence of motor complications, and the Mini Mental State examination (MMSE score)] of these patients were retrieved. Moreover, treatment data (dopaminergic medication selec-tion, combination, and dosage, as well as the levodopa equivalent daily dose LEDD) were evaluated.

Due to the relative benign nature of motor deficits in *PRKN* and *PINK1* carriers, initial treatment included low to moderate doses of dopaminergic agonists like pramipexole or rotigotine and rasagiline. As the disease progressed, certain patients were initiated with low L-dopa doses that were further adjusted following two principles: (1) to be sufficient in order to enable patients maintain a satisfactory motor function, and (2) to avoid the early emergence of motor complications (fluctuations and dyskinesias) known to be triggered by higher L-Dopa doses, notably in early-onset PD cases. Amantadine was selected for patients not responding favorably to L-Dopa or for those with motor complications.

We have further compared the LEDD results of patients in the recessive gene variants cohort (*N* = 16) with a sex-matched (8 M/8 F) idiopathic PD cohort from the ‘’Thalis’’ database, using a univariate analysis of co-variance (ANCOVA) with age and disease duration as covariates. The statistical analyses were performed using commercially available software (SPSS, Version 29.0). The present study was conducted in agreement with the principles of the Declaration of Helsinki. Signed informed consent was obtained from all participants recruited. The study was approved by the Scientific Board of Eginition hospital (Committee approval: Protocol Number 658/06/12/2020).

## 3. Results

The epidemiological and basic clinic data of PD carriers of pathogenic variants of recessive genes in our cohort are shown in Table 1. In total, five patients of the recessive gene carriers cohort had a positive family history for PD (three *PRKN* and two *PINK1* mutation carriers). *PRKN* PD carriers (6 M/7 F) had an average age of 50.4 ± 10.9, an average age at onset of 37.6 ± 7.6, and a disease duration of 14.3 ± 9.2 years. In *PINK1* carriers (2 M/1 F), the mean age was 64.7 ± 11.4, average age at onset 35 ± 3, and disease duration 29.7 ± 9.5 years. The H&Y score was relatively low even after decades of disease in carriers of both genes (2 ± 0.7 for *PRKN* carriers and 2.33 ± 0.58 for *PINK1* carriers). Similarly, the MMSE score was excellent after many years of disease (29.54 ± 0.52 for *PRKN* and 27.33 ± 0.71 for *PINK1* carriers). As far as the presence of motor complications is concerned, 9/13 of *PRKN* mutation carriers exhibited motor fluctuations and/or dyskinesias during their disease course. The same was true for 2/3 of *PINK1* mutation carriers.

Regarding treatment, the average levodopa equivalent daily dose (LEDD) in *PRKN* carriers was 806.8 ± 453.5 (range 152–1810). Similarly, in *PINK1* carriers the average LEDD was 765 ± 96.6 (range 660–850). Detailed treatment data for each participant are shown in Table 2. As far as therapeutic options are concerned, the majority of patients responded to low/moderate doses of levodopa and were prone to the development of motor fluctuations and dyskinesias. A levodopa dosage lower than 400 mg/day was sufficient for 6/13 patients. Nevertheless, certain patients (3/16) could not tolerate even low levodopa doses, and alternative medication schemes like dopamine agonists and/or amantadine were selected. The response to dopamine agonists (DAs) was often favorable both as initial and longitudinal therapy, although side effects occasionally arose (7/16 patients needed to discontinue or reduce the dosage of DAs due to impulse control disorders or psychiatric manifestations). We have to underline the fact that 2/13 *PRKN* patients had clear-cut disabling psychotic features during their disease course and a third one manifested milder psychotic symptoms. All three had to receive antipsychotic treatment. These patients were first treated with quetiapine with a dosage range of 25–100 mg/d. All of them had an initial favorable response but during disease progression two out of three had to receive increasing doses of quetiapine up to 300 mg/d which was later replaced by olanzapine (up to 5 mg/d) and finally by clozapine 25–50 mg/d. In such cases, psychosis occurred in the absence of dementia and was probably more linked to dopaminergic treatment, especially dopaminergic agonists. It may also manifest as an isolated delusional syndrome [29]. Psychotic symptoms or treatment were not reported among *PINK1* carriers in our cohort.

Rasagiline was also a useful add-on therapy (5/16). A very high number of carriers, 9/13 *PRKN* and 1/3 *PINK1,* were treated with amantadine successfully, and this also applied to patients who could not tolerate levodopa or DAs. In some cases, amantadine had an especially good response in cases with bizarre gait patterns.

Due to the relatively mild symptomatology of patients in our cohort, there was no need for device-aided treatments (either an s.c. apomorphine/intestinal levodopa–carbidopa gel pump or deep brain stimulation (DBS)).

As far as a direct comparison between the recessive gene PD cohort vs. idiopathic PD from the ‘’Thalis’’ study is concerned, after adjusting for age and disease duration, LEDD showed a tendency to be lower in the recessive gene carriers (798.9 ± 407.5) compared to idiopathic PD (880.4 ± 389.1), but the difference was not statistically significant (*p* = 0.196). Interestingly, 16/16 patients in the idiopathic PD group had been treated with L-Dopa vs. 13/16 in the recessive gene PD cohort while 3/16 patients in the latter were doing well without any L-Dopa treatment.

## 4. Discussion

The heterogeneity in the clinical features of PD has been elucidated thanks to large patient cohorts which have undergone deep phenotyping [2,7]. However, as far as treatment is concerned, the approach is uniform and predominantly symptomatic. Research evidence from targeted therapies for monogenic forms of PD aiming at neuroprotection may pave the way for new mechanism-based interventions for genetic PD forms and also for the more common idiopathic PD. An ameliorated stratification of patients might also support symptomatic treatments by predicting treatment efficacy and long-term adverse effects.

The clinical picture and course vary depending on the exact genetic cause and the specific mutation, but there is also a variability even within families with the same mutation [2]. The genetic diagnosis of the disease is important in these cases not only for genetic counseling and the assessment of patient prognosis, but also because it can be taken into account for specific treatment options, including device-aided therapies (like deep brain stimulation (DBS) or levodopa–carbidopa enteric gel pumps). Furthermore, such patients could enroll in emerging neuroprotective clinical studies that are applied to specific genetic forms of the disease, in the context of pharmacogenomics [30]. With the gradual establishment of personalized treatment, the therapeutic approach in genetic PD forms might differ substantially as compared to idiopathic PD [31].

A very important issue to address is individual therapy optimization. Treatment with antiparkinsonian drugs is associated with the development of complications, such as levodopa-induced fluctuations and dyskinesias, hallucinations, and excessive daytime sleepiness. Carriers of specific genetic forms may be particularly susceptible to the development of some of these adverse drug effects. To our knowledge, there are a limited number of studies assessing dopaminergic medication schemes in cohorts of genetic PD patients carrying mutations in recessive genes. Although previous review articles on recessive PD genes provide information on treatment in such patients, real-world data from clinical cohorts are sparse [32,33]. Most data derive from case reports or small case series. The majority of existing literature on precision treatment according to the genetic background has focused either on disease-modifying treatments or device-aided therapies. Notably, more research has taken place regarding device aided treatments in patients with genetic PD that is not recessive (Glucocerebrosidase gene (*GBA1*) or Leucine-Rich Repeat Kinase 2 (*LRRK2*) carriers) [31].

A new study evaluated the impact of levodopa–carbidopa intestinal gel infusion (LCIG) on 56 PD patients and genetic mutations were confirmed upon genetic testing in 9/56 (15%) (5 *GBA1*, 2 Alpha-synuclein (*SNCA*), 1 *LRRK2*, 1 *PRKN*). Patients who underwent LCIG demonstrated an improvement of motor complications. This was also true for carriers of genetic mutations. No effect of the presence of pathogenic mutations regarding motor or cognitive functions could be observed [34].

In our cohort, lower LEDD doses were efficient even in patients with a very long disease duration, while certain patients were doing well without any levodopa treatment decades after disease initiation. Despite a trend for lower LEDD in the *PRKN*/*PINK1* group vs. idiopathic PD, the lack of a statistical significant difference could be attributed to the small number of participants or to the pronounced variability of LEDD in both groups. Other therapeutic options had been selected. DAs can be used as a first- and main-line treatment regimen if well tolerated. Rasagiline is quite effective either as an initiation or an add-on therapy in patients with recessive PD forms. It furthermore appears that amantadine represents an attractive therapeutic option in PD with mutations in recessive genes, since it was generally effective and well tolerated. As we have pointed out above, carriers of mutations in recessive genes in our cohort generally had a mild clinical course in terms of motor and cognitive function (low H&Y score and excellent MMSE score even after long disease duration). Moreover, our results on the absence of dementia are compatible with literature concerning *PRKN* and *PINK1* mutations [2,7] although there are some exceptions notably for *PINK1* carriers [35,36]. We should also mention the occurrence of psychotic symptoms in a minority of patients which can be isolated, triggered by dopamine agonists treatment, and irrespective of general cognitive decline. According to our results, an impressive observation regarding treatment requirements is that a proportion of PD patients (3/13) carrying *PRKN* mutations had an excellent response to dopamine agonists and had not been started on L-Dopa therapy even after 20 years since disease onset. The lack of need for L-Dopa therapy supports the relatively benign nature of recessive gene-related PD [5].

Amantadine administered as monotherapy in the early stages of PD produces a moderate improvement, which appears in a few days and concerns all the symptoms of the disease. Moreover, in advanced PD, amantadine has been shown to be effective in reducing dyskinesias based on its N-methyl-D-aspartate (NMDA) receptors blocking property. It is notable that in our cohort, 10/16 patients were treated successfully with amantadine, providing evidence of its role in either early or advanced recessive gene-related PD.

Furthermore, the lack of device-aided approaches in our cohort might also be compatible with relatively low doses of L-Dopa overall and the relatively benign nature of *PRKN*- or *PINK1*-related PD even in terms of motor symptoms. However, a confounding factor is the difficulty of accessibility to DBS in Greece, which is the main device-aided therapy in young subjects. The latter may also be a contributing factor to the lack of such subjects in our cohort. Comparatively, in the review of Over et al., 20 out of 1002 PD patients carrying *PRKN* mutation and 3 out of 151 *PINK1* mutation carriers had undergone DBS [32].

According to previously published data, PD patients harboring *PRKN* mutations require a low LEDD for excellent control of motor signs, but despite receiving markedly low doses of levodopa, they exhibited the frequent development of motor complications and dyskinesias from early disease stages. Based on the previously mentioned features of PD patients carrying *PRKN* mutations, starting with a low LEDD and selecting dopaminergic agonist treatment rather than levodopa is recommended in the early stages [24,37]. However, in some studies, dopamine agonists are less effective on motor symptoms and do not delay the onset of motor fluctuations and dyskinesias compared to levodopa in the long run. Despite the absence of many other non-motor symptoms, neuropsychiatric disturbances are often present and severe in *PRKN* mutation carriers, mainly impulsive–compulsive behaviors (including gambling, compulsive buying, binge eating, and sexual behaviors or excessive interest in hobbies) [38]. Risk factors for therapy-related impulsive behavior include a novelty-seeker impulsive personality and the patient’s demographic profile including young age, male sex, and previous neuropsychiatric symptoms. These clinical characteristics should prompt for careful selection of dopaminergic therapy doses, as well as frequent monitoring for impulse-control disorders and behavioral disturbances. Bohlega and co-authors reported good motor and non-motor outcomes in a heterozygous *PRKN* mutation carrier with long follow-up [39], while Foltynie and co-authors describe two *PRKN* patients with compulsive behavior, but the outcomes were not specified [40].

We were able to largely replicate the observations of previous publications on recessive PD forms. A recent review evaluated the current landscape of treatments of monogenic PD forms including *PRKN* and *PINK1* [32]. In this review study, 1002 PD patients harbored biallelic *PRKN* mutations and 54.2% were receiving L-Dopa. The majority reported a favorable response to levodopa (average dose of 490 mg/d). However, a few patients had a poor response to the aforementioned treatment. Side effects included motor complications (dyskinesias and fluctuations) and dopamine dysregulation or psychotic symptoms mostly due to DAs. DBS surgery was reported in 20 *PRKN* mutation carriers, and a good clinical response was reported in all patients [32].

Regarding *PINK1* in the same review [32], 151 patients with biallelic *PINK1* mutations were assessed. In total, 75.5% of carriers were on L-Dopa treatment. Most *PINK1* mutation-harboring PD patients had a favorable response (97.9%). The average dose was 350 mg/day. Adverse effects also included mostly motor complications (fluctuations and dyskinesias). Three patients underwent DBS with a favorable outcome [32].

In the era of personalized treatment, the therapeutic approach in PD needs to be more targeted [32,33]. Genetic PD forms represent a novel field where precision medicine could be applied [37]. Recessive forms of PD in particular share unique features, and patients may benefit significantly from individualized treatments [20,37]. Better stratification strategies leading to precision medicine approaches pave the way for the improvement of symptomatic treatment and the ability to provide more patient-targeted care. Studies such as ours, focusing on the peculiarities of current pharmacological treatments in autosomal recessive genetic PD, may be useful to provide the framework upon which more specific disease-modifying therapies may be applied in the future.

**Figure 1:**
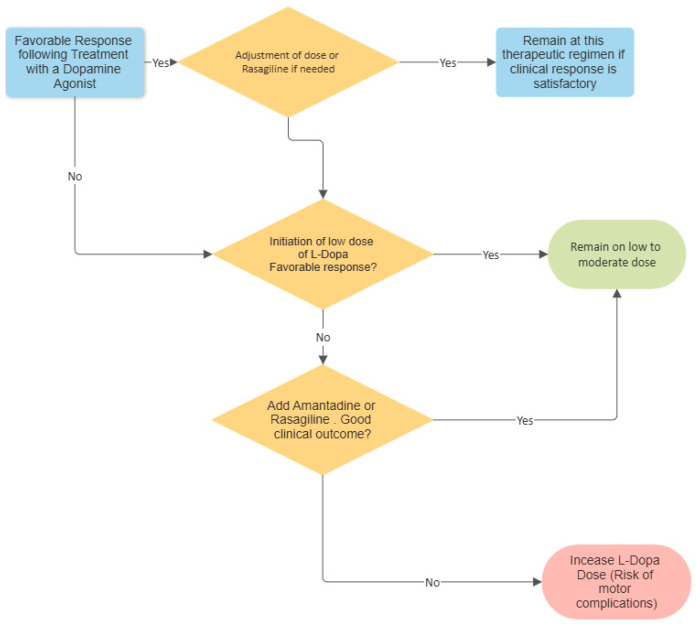
Flow process chart regarding the therapeutic regimen in PD patients carrying pathogenic mutations in recessive genes.

Our single-center study adds to the body of evidence indicating that autosomal recessive PD due to *PRKN* or *PINK1* mutations has a relatively benign nature and demonstrates a uniformly good therapeutic response to oral pharmacological treatment, despite prolonged disease duration. The study also provides evidence that treatment with amantadine may be particularly effective in this population. Lack of treatment with levodopa 10 years or more after PD diagnosis in a relatively young patient should raise suspicions about the existence of autosomal recessive PD (Figure 1).

## 5. Conclusions

In the era of precision medicine and individualized treatment, the therapeutic approach in recessive PD gene carriers might differ as compared to idiopathic PD. Lower LEDD doses were efficient even in patients with a very long disease duration, while a few patients were doing well without any levodopa treatment decades after disease initiation. DAs could be used as a first and main line treatment regimen if well tolerated. It furthermore appears that amantadine represents an attractive therapeutic option in PD with mutations in recessive genes, since it was generally effective and well tolerated. Finally, according to the literature data, in progressed disease stages most carriers of mutations in recessive PD genes have a favorable response to device-aided therapies.

## Figures and Tables

**Table 1:** Demographic and basic clinical data of PD patients carrying pathogenic or likely pathogenic variants in recessive PD-related genes.

	Age	Sex	Age at Onset	Duration (Years)	H&Y	Motor Complications	MMSE
PRKN 1	56	F	40	16	2	Absent	30
PRKN 2	67	F	47	20	3	Present	29
PRKN 3	41	F	29	12	1	Absent	30
PRKN 4	65	F	45	40	3	Present	30
PRKN 5	48	M	42	6	2	Present	29
PRKN 6	66	F	45	21	3	Absent	30
PRKN 7	48	M	40	8	1	Present	29
PRKN 8	40	F	32	8	2	Present	30
PRKN 9	35	M	29	6	1	Absent	29
PRKN 10 (Het)	57	F	44	13	2	Present	29
PRKN 11 (Het)	40	M	24	16	2	Present	29
PRKN 12(Het)	42	M	31	11	2	Present	30
PRKN 13(Het)	50	M	41	9	2	Present	30
PINK1 1	52	M	32	20	3	Absent	29
PINK1 2	74	F	35	39	2	Present	28
PINK1 3	68	M	38	30	2	Present	25

H&Y: Hoehn and Yahr Scale, MMSE: Mini Mental State Examination, Het: Heterozygote.

**Table 2:** Treatment data of PD patients carrying pathogenic or likely pathogenic variants in recessive PD-related genes.

	Age	Sex	Duration (Years)	LEDD	L-Dopa	COMT Inhibitors	Dopa-Agonists	Rasagiline	Amantadine
PRKN 1	56	F	16	670	150 mg	-	Ropinirole 6 mg	-	400 mg
PRKN 2	67	F	20	1340	900 mg	-	Rotigotine 8 mg	-	200 mg
PRKN 3	41	F	12	320	-	-	Ropinirole 6 mg	-	200 mg
PRKN 4	65	F	40	793	250 mg	Entacapone 1000 mg	Ropinirole 8 mg	1 mg	200 mg
PRKN 5	48	M	6	905	600 mg	-	Pramipexole 1.05 mg	-	200 mg
PRKN 6	66	F	21	320	-	-	Ropinirole 6 mg	-	200 mg
PRKN 7	48	M	8	720	200 mg	-	Rotigotine 4 mg	-	400 mg
PRKN 8	40	F	8	1810	1600 mg		Pramipexole 2.1 mg	-	-
PRKN 9	35	M	6	660	200 mg	-	Ropinirole 8 mg	1 mg	200 mg
PRKN 10 (Het)	57	F	13	152	100 mg	-	Pramipexole 0.52 mg	-	-
PRKN 11 (Het)	40	M	16	833	350 mg	Entacapone 600 mg	-	1 mg	300 mg
PRKN 12(Het)	42	M	11	705	600 mg	-	Pramipexole 1.05 mg	-	-
PRKN 13(Het)	50	M	9	1260	1050 mg		Pramipexole 2.1 mg	-	-
PINK1 1	52	M	20	660	-	-	Ropinirole 8 mg	1 mg	400 mg
PINK1 2	74	F	39	785	500 mg	Entacapone 800 mg	Rotigotine 4 mg	-	-
PINK1 3	68	M	30	850	750 mg	-	-	1 mg	-

LEDD: levodopa equivalent daily dose; COMT: catechol-O-methyltransferase.

## Data Availability

The data supporting the findings of this study are available upon reasonable request from any qualified investigator.
